# Physical Stress, Not Biotic Interactions, Preclude an Invasive Grass from Establishing in Forb-Dominated Salt Marshes

**DOI:** 10.1371/journal.pone.0033164

**Published:** 2012-03-14

**Authors:** Qiang He, Baoshan Cui, Yuan An

**Affiliations:** 1 State Key Laboratory of Water Environmental Simulation, School of Environment, Beijing Normal University, Beijing, China; 2 School of Agriculture and Biology, MOA Key Laboratory of Urban Agriculture (South), Shanghai Jiao Tong University, Shanghai, China; Duke University, United States of America

## Abstract

**Background:**

Biological invasions have become the focus of considerable concern and ecological research, yet the relative importance of abiotic and biotic factors in controlling the invasibility of habitats to exotic species is not well understood. *Spartina* species are highly invasive plants in coastal wetlands; however, studies on the factors that control the success or failure of *Spartina* invasions across multiple habitat types are rare and inconclusive.

**Methodology and Principal Findings:**

We examined the roles of physical stress and plant interactions in mediating the establishment of the smooth cordgrass, *Spartina alterniflora*, in a variety of coastal habitats in northern China. Field transplant experiments showed that cordgrass can invade mudflats and low estuarine marshes with low salinity and frequent flooding, but cannot survive in salt marshes and high estuarine marshes with hypersaline soils and infrequent flooding. The dominant native plant *Suaeda salsa* had neither competitive nor facilitative effects on cordgrass. A common garden experiment revealed that cordgrass performed significantly better when flooded every other day than when flooded weekly. These results suggest that physical stress rather than plant interactions limits cordgrass invasions in northern China.

**Conclusions and Significance:**

We conclude that *Spartina* invasions are likely to be constrained to tidal flats and low estuarine marshes in the Yellow River Delta. Due to harsh physical conditions, salt marshes and high estuarine marshes are unlikely to be invaded. These findings have implications for understanding *Spartina* invasions in northern China and on other coasts with similar biotic and abiotic environments.

## Introduction

Biological invasions have recently become the focus of considerable concern and ecological research [1]. Although the ecological consequences of biological invasions have been well documented [2], [3], the mechanisms that control invasions and the factors that mediate the susceptibility or resistance of habitats to exotic species invasions, i.e., invasibility [2], [4], remain unclear [5].

The invasibility of habitats to a specific exotic species after propagules arrive is potentially determined by several abiotic and biotic factors [2], [4], [6]. Many studies have investigated the role of either the physical environment or properties of native communities in determining the invasibility of habitats, yet studies investigating the relative importance of both these factors across habitat types are only a few [6], [7], [8], [9]. Here, we examine the roles of physical stress and plant interactions in mediating the establishment of smooth cordgrass, *Spartina alterniflora*, across estuarine and non-estuarine habitats in northern China.

Estuarine and coastal systems are among the most heavily invaded systems in the world [10]. *S. alterniflora* and several other species in the widely invasive genus *Spartina* have successfully invaded coastal wetlands in China and across the globe [11], [12], [13]. These invasions can dramatically impact the structure and function of invaded wetlands [12], [14]. The morphology, physiology (e.g. [15], [16], [17], [18], [19], [20]) and invasiveness (e.g. [12], [21], [22], [23], [24], [25], [26]) of *Spartina* species have all been well documented. However, the invasibility of habitats to *Spartina*, which is of particular importance for predicting future *Spartina* invasions [6], [8], [25], [26], [27], needs to be further examined.

*Spartina alterniflora* (hereafter referred to as cordgrass), introduced to China in 1979 [22], [28] was suggested to be capable of invading both high salinity coastal marshes and low salinity estuaries [22]. Previous studies focused on the impact of cordgrass invasions on the structure and function of native communities (see [28], [29] for reviews). A previous study [8] used pot experiments to investigate the interactive effects of plant competition and environmental gradients on cordgrass invasions in the Yangtze estuary in southern China, but such experiments were seldom done in the field, particularly in northern China, which differs from southern China in both the physical environment and community structure. In contrast to southern coastal marshes dominated by perennial grass species, most northern salt marshes are dominated by the annual succulent *Suaeda salsa*, a highly stress-tolerant but competitively inferior species [28], [30], [31].

In this study, we focus on the invasibility of habitats to cordgrass in the Yellow River Delta in northern China by testing the hypothesis that cordgrass is prevented from invading hypersaline marshes by harsh physical conditions rather than interspecific competition. The habitats examined occur in different zones in both estuarine and non-estuarine systems. We first describe the physical environment and vegetation in these habitats, and then examine how cordgrass performance is mediated by physical stress and plant interactions in different habitats using transplant and common garden experiments.

## Materials and Methods

### Study sites

**Figure 1:**
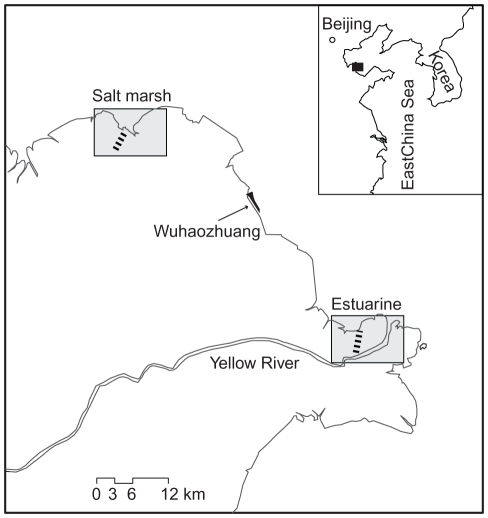
Map showing study sites in the Yellow River Delta, northern China. Cordgrass was artificially planted at Wuhaozhuang in the 1980s, and has invaded the estuarine site. Cordgrass was collected at Wuhaozhuang and transplanted into different zones (along the dotted lines) at the salt marsh and estuarine sites.

Field work was conducted in the Yellow River Delta National Nature Reserve, northern China (Figure 1). We got permits from the managers of the reserve to enter any parts of the reserve, do field survey and manipulative experiments, and collect soil and plant samples. No specific permissions were required for the study sites/activities, and no endangered or protected species were involved in the present study. An estuarine site and a non-estuarine salt marsh site in the reserve were used. The estuarine site is a low salinity system with significant freshwater input, particularly during the summer. In contrast, the salt marsh site is a high salinity system dominated by seawater input. Both sites experience irregular, semidiurnal tides and a warm-temperate climate. Substrate salinity, one of the most limiting physical stressors in coastal habitats, increases with elevation at both sites [31], [32].

At each site, three zones were defined based on elevation and vegetation: mudflat, low marsh and high marsh. At the estuarine site, cordgrass has recently colonized the mudflats and is in the early stages of invasion [32]. *Suaeda salsa*, an annual halophyte, dominates the low and high marsh zones. Salt pans and *Tamarix chinensis,* a shrubby halophyte, are also common at upper elevations, while patches of *Phragmites australis* are found in the low marsh zone. Other plants observed at the estuarine site include *Salicornia europaea*, *Scirpus* spp. and *Juncus* spp. [32].

Cordgrass has not invaded the mudflat zone at the salt marsh site, which remains unvegetated (Qiang He, personal observation). The low and high marsh zones at the salt marsh site are similarly dominated by *S. salsa* and sparse *T. chinensis* at upper elevations, but overall vegetation cover is much lower and hypersaline salt pans without vegetation are prevalent [31]. Few other plant species occur there.

### Quantification of physical stresses and vegetation

To quantify physical stresses at the two sites, we randomly collected eight soil samples (5.05 cm diameter×5 cm depth) in each of the three zones at each site in July 2010. At the salt marsh site where the cordgrass transplant experiment was initiated in 2009, soil samples were also collected in July 2009. As our previous studies [31], [32] have documented seasonal variation in physical stresses at these sites, we simply quantified relative differences among zones and sites, and did not sample on multiple dates.

**Table 1:** Physical stresses and vegetation cover in different zones at the salt marsh and estuarine sites.

	Salt marsh site			Estuarine site			*P*		
	High marsh	Low marsh	Mudflat	High marsh	Low marsh	Mudflat	Zone	Site	Site*Zone
Salinity (g/kg)									
2009	160.6(17.1)	109.0(3.1)	46.9(2.3)	-	-	-	-	-	-
2010	211.29(12.8)	76.64(3.4)	30.68(1.5)	164.68(9.7)	45.1(0.6)	29.7(0.8)	<0.01	<0.01	<0.01
Moisture (%)									
2009	18.9(0.3)	23.1(0.2)	22.3(0.2)	-	-	-	-	-	-
2010	17.4(0.7)	22.6(0.3)	23.4(0.4)	19.0(0.6)	24.5(0.5)	22.2(0.4)	<0.01	0.30	0.02
Flooding (%)	5–20	30–50	70–90	5–15	40–60	80–90	-	-	-
Vegetation cover (%)	3.87(1.5)	10.47(2.0)	-	83.67(1.6)	38.67(2.7)	-	0.17	<0.01	<0.01

Data are means ± 1SE. Sample sizes are 8 for salinity, moisture, and 15 for vegetation cover. Flooding frequency (shown as a range) was estimated based on previous studies and field observations.

Statistical analyses were conducted using generalized linear models. Gamma regressions with log link were used for salinity and moisture while a Poisson regression with log link was used for vegetation cover. -, no data. Flooding indicates the percentage of days flooded in a year.

Soil cores were oven-dried and weighed to determine soil moisture. Dried soil was mixed with a known volume of deionized water. The salinity of the supernatant was measured after 24 hours using an electronic meter, and the original soil pore water salinity was calculated based on the initial water content of the core [33]. We estimated flooding frequency for each zone based on long-term field observations at each site (see [31], [32]).

Vegetation in the low and high marsh zones at each site was quantified in August 2010. We randomly located 15 quadrats (1×1 m) in each habitat at each site and visually estimated percentage vegetation covers (mostly *S. salsa* plants).

Differences in abiotic factors and vegetation cover among zones and sites in 2010 were analyzed using generalized linear models, as these data did not meet the assumptions of ANOVA. Generalized linear models are a widely accepted alternative test when data cannot be transformed to meet the assumptions of ANOVA [34]. We used Gamma distribution with log link for soil salinity and moisture, and Poisson distribution with log link for vegetation cover data. Differences in soil salinity and moisture between 2009 and 2010 at the salt marsh site were examined using one-way ANOVAs, or non-parametric Wilcoxon tests if the data did not meet the assumptions of ANOVA after transformation.

### Field transplant experiments

To determine the invasibility of various habitats to cordgrass in the Yellow River Delta, we transplanted cordgrass replicates into the mudflat, low marsh and high marsh zones at the salt marsh site in May 2009. As the mudflat and the high marsh zones had little vegetation, the experimental design was not fully factorial with neighboring vegetation. We excavated a number of substrate blocks (16×16×20 cm) containing emerging cordgrass tillers at Wuhaozhuang where cordgrass was introduced in the 1980s (Figure 1). These substrate blocks were immediately transplanted into the mudflat (*n* = 15), low marsh (*n* = 16) and high marsh (*n* = 12) zones at the salt marsh site. We mainly aimed to compare cordgrass performance among different zones at this site, and did not transplant cordgrass back to Wuhaozhuang as controls. The transplants were watered with fresh water every other day for a week to reduce transplant shock. Half of the transplants in the low marsh were assigned to neighbor-removal treatments. Neighbors surrounding the transplants were removed by clipping a 25 cm-radius border at the soil surface every other week as needed. Neighbors adjacent to the remaining transplants were unmanipulated. Neighbor removal treatments were maintained semi-monthly as needed throughout the growing season. We did not conduct neighbor removal treatments for the high marsh transplants, as native plants were very sparse in the high marsh with only a few scattered *S. salsa* and stunted *T. chinensis*. Transplants were maintained for two growing seasons. The number of stems, inflorescences and maximum plant height of each transplant were determined at the end of the growing season in September 2009 and 2010. Above-ground biomass was harvested in September 2010, oven-dried at 60°C for 48 hours, and weighed.

**Figure 2:**
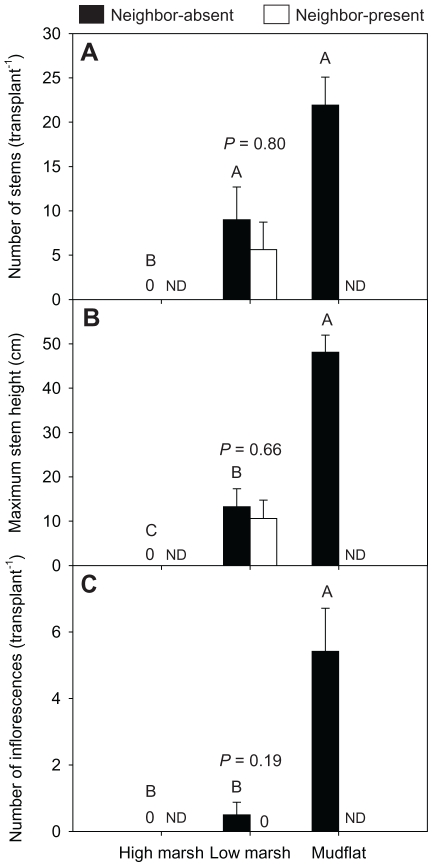
Cordgrass performance in different zones at the salt marsh site in 2009. (A) number of stems (B) maximum plant height and (C) number of inflorescences. Data are means + 1SE. ND indicates no data. Bars sharing a capital letter were not significantly different from one another (nonparametric multiple comparisons, Steel-test). *P*-values from ANOVAs or Wilcoxon tests investigating the effect of neighbors are indicated above the bar group for low marsh transplants.

**Figure 3:**
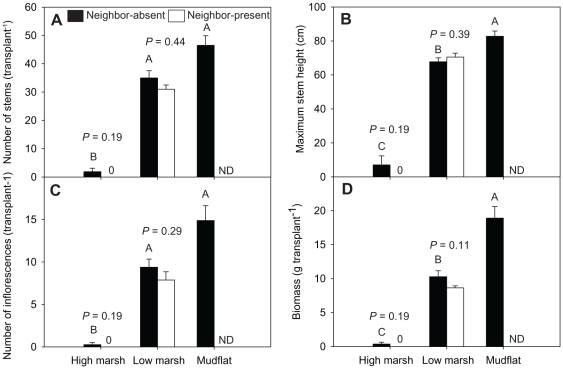
Cordgrass performance in different zones at the estuarine site in 2010. (A) number of stems (B) maximum plant height (C) number of inflorescences and (D) biomass. Data are means + 1SE. ND indicates no data. Bars sharing a capital letter were not significantly different from one another (nonparametric multiple comparisons, Steel-test). *P*-values from ANOVAs or Wilcoxon tests investigating the effect of neighbors are indicated above each bar group for low marsh and high marsh transplants.

We conducted a second transplant experiment at the low salinity estuarine site in 2010 to investigate additional habitats with varying abiotic stress. Substrate blocks (same size and origin as 2009) with emerging cordgrass tillers were excavated and transplanted into the mudflat (*n* = 8), low marsh (*n* = 16) and high marsh (*n* = 16) zones. These transplants were watered for the first week, and half of the transplants in the low and high marsh zones were assigned and maintained as neighbor-removal treatments as described above. Neighbors adjacent to the remaining transplants were unmanipulated. In September 2010, the number of stems, inflorescences and maximum plant height of each transplant were determined, above-ground biomass harvested, oven-dried and weighed.

The effect of zone on cordgrass performance was analyzed using non-parametric multiple comparisons (Steel test, [35]), as high mortality of cordgrass transplants made the data highly skewed. The effect of native vegetation was examined using one-way ANOVAs or non-parametric Wilcoxon tests, depending on whether the assumptions of ANOVA were met.

### Common garden experiment

To examine the role of flooding on cordgrass performance in the Yellow River Delta salt marshes, we conducted a common garden experiment. In May 2010, substrate blocks (16×16×20 cm) with emerging cordgrass tillers were excavated at Wuhaozhuang and transplanted into a common garden at our field station. Two cordgrass blocks were planted in 0.9×0.9 m tanks (*n* = 12) filled with soil collected from a salt marsh near the common garden. The soils were mixed thoroughly to ensure homogeneity among plots prior to treatments. The soil in each plot was approximately 30 cm deep. We did not plant *S. salsa* in these artificial plots, as our 2009 field experiment suggested that *S. salsa* had no effect on cordgrass performance. Plots were watered with seawater every other day for the first two weeks to reduce transplant shock. We then randomly assigned half of the plots to one of two different flooding regimes: every other day and weekly, to simulate the natural flooding of the low and high marsh zones, respectively. Seawater collected from the nearest tidal channel (filled with seawater at high tide) was used for flooding treatments. Flooding treatments were done by pouring ∼ 25 L seawater into each plot in late afternoons and letting it drain through a 6 cm hole on one side of the plot. In late September 2010, we determined the performance of cordgrass transplants in each plot by counting the number of stems and measuring maximum plant height. Above-ground biomass in each plot was harvested, oven-dried and weighed. The effect of flooding on cordgrass performance was examined using one-way ANOVAs or non-parametric Wilcoxon tests, depending on whether the assumptions of ANOVA were met.

## Results

### Physical stresses and vegetation

Physical stresses and vegetation varied between zones and sites (Table 1). For both the salt marsh and estuarine sites, soil salinity was lowest in the mudflat, intermediate in the low marsh and highest in the high marsh (Table 1). Soil salinity in the low and high marsh zones was much higher at the salt marsh site than at the estuarine site, but soil salinity in the mudflat did not differ between sites in 2010. Soil moisture was much higher in the low marsh and the mudflat than in the high marsh at both sites (Table 1). Flooding frequency decreased from the mudflat to the high marsh at both sites (Table 1). Although the overall pattern of soil salinity across zones at the salt marsh site was the same between 2009 and 2010, there were some significant differences (Table 1). Soil salinity was higher in the low marsh and the mudflat but much lower in the high marsh in 2009 than in 2010 (*P*<0.05). Vegetation cover (mostly *S. salsa*) was significantly higher in the high marsh than in the low marsh at the estuarine site (Table 1). However, vegetation cover was very low in both the low and high marsh zones at the salt marsh site (Table 1).

**Figure 4:**
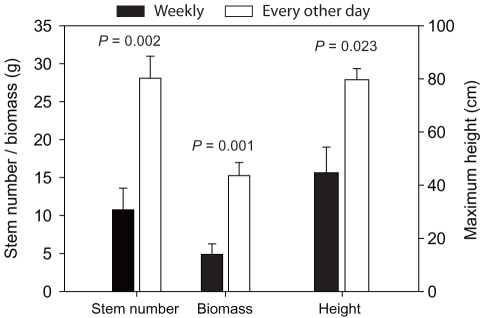
Cordgrass performance in different flooding treatments in the common garden experiment. (A) number of stems and maximum plant height and (B) biomass. Data are means + 1SE (*n* = 6). *P*-values from ANOVAs or Wilcoxon tests are indicated.

### Field transplant experiments

At the salt marsh site, cordgrass transplants grew well in the mudflat in September 2009 (after one growing season), but their growth decreased with increasing elevation (Figure 2). The number of stems, maximum plant height and number of inflorescences were all highest in the mudflat. In the low marsh, a few cordgrass transplants survived, but their growth and reproduction were much lower than those in the mudflat (Figure 2b and c). None of the cordgrass transplants survived in the high marsh. In September 2010, after two growing seasons, however, most cordgrass transplants in all of the three habitats died. The number of stems, biomass, number of inflorescences and maximum plant height for the few surviving transplants were all very low, and did not differ among zones and neighbor treatments (Table S1).

At the estuarine site, cordgrass transplants also performed best in the mudflat and worst in the high marsh (Figure 3). Number of stems, maximum plant height, number of inflorescences and biomass of cordgrass transplants were all highest in the mudflat. In the low marsh, most cordgrass transplants also survived and performed relatively well, although maximum plant height and biomass were significantly lower in the low marsh than in the mudflat (Figure 3b and d). Most of the cordgrass transplants died in the high marsh, regardless of neighbor treatments. None of the fitness parameters differed significantly between neighbor-removal and neighbor-present treatments in the low or high marsh at the estuarine site (Figure 3).

### Common garden experiment

Cordgrass transplants grew much better when flooded every other day than when flooded weekly (Figure 4). Cordgrass stem number, maximum plant height and biomass in the every other day flooding treatment were much higher than those in the weekly flooding treatment (Figure 4).

## Discussion

Our results reveal that cordgrass transplants generally perform well in mudflats and low estuarine marshes with low salinities and frequent flooding. They cannot establish, however, in high estuarine marshes and salt marshes where soils are hypersaline and seldom flooded. We found neither a positive nor negative effect of native vegetation on cordgrass, even in estuarine marshes where neighbor effects were expected (due to abundant native vegetation and the well performance of the transplants). These results suggest that physical stress rather than competition from native plants are likely to control cordgrass invasions in the Yellow River Delta.

Despite the well-documented ability of *Spartina* species to tolerate salt and flooding stress [18], [20], [36], we found that hypersaline habitats, particularly at upper elevations, prevented the establishment, growth and sexual reproduction of cordgrass transplants. This result is consistent with a previous study which suggests that salinity is a critical physical stressor that restricts *S. anglica* invasions in the Pacific Northwest, USA [6]. The performance of *Spartina* species is generally believed to decrease with increasing salinity [18], [19], [21], [37]. The results from our field and common garden experiments suggest that frequent flooding favors the establishment and growth of cordgrass, results that are consistent with previous studies reporting increased biomass of cordgrass under frequent flooding regimes [6], [8], [36], [38]. This may be due to infrequent flooding leading to elevated salt stress by soil heating and evaporation of pore water [6], [25], [26], [39], [40]. Although we did not quantify salinity differences between weekly and every other day flooding treatments in the common garden experiment, the accumulation of salt on the soil surface in weekly flooding treatments was visually striking, suggesting that increased salt stress was responsible for the lower performance of cordgrass there.

We found no evidence that competition with native species mediates the performance of cordgrass transplants in the Yellow River Delta, despite the paradigm that lower competitive ability restricts cordgrass to lower zones in its native ranges [33], [36], [41]. At the salt marsh site, the lack of positive or negative interactions between native vegetation and the transplanted cordgrass can be due to the low abundance of native vegetation. In the low estuarine marsh where both cordgrass and native vegetation grow well, however, the lack of a neighbor effect should be a result of no interactions, instead of a result of having no neighbors; in the high estuarine marsh where the native vegetation grow well while cordgrass performs very poorly, no neighbor effect can be a result of the lack of cordgrass. Because the estuarine marshes are dominated by stunted *S. salsa*, its ability to reduce soil salinity by shading is minor [32], [42], and as a result, *S. salsa* has little positive effect on cordgrass growth and persistence. In a previous study, Dethier and Hacker (2005) [6] also found that species interactions had no effect on *S. anglica* performance. In addition, although it has been suggested that the outcome of interactions between native *P. australis* and cordgrass can change along environmental gradients and that native plants can outcompete cordgrass under some conditions [8], we found no evidence that *S. salsa* can outcompete cordgrass at any of the habitats we examined.

Two limitations of our experiments should be noted. First, we focused on how the establishment and reproduction of transplanted cordgrass ramets with rhizomes varied among different habitats, as the documented large-scale invasions of this species in China have been shown to rely mainly on human-aided transplanting [28], although after plantation this species expands at a local range by both sexual and vegetative reproduction [43], [44]. Nevertheless, as *Spartina* species can be more susceptive to physical stress and species interactions at the germination or seedling establishment stage than it is at the clone stage [6], [37], their dynamics, particularly in hypersaline conditions where seedling recruitment and growth are often limited, should be mainly controlled by vegetative growth [37], [45]. Second, in the field experiments, we did not establish transplant controls at Wuhaozhuang where cordgrass were excavated. Transplanting may affect the vigor of species. But as the physical conditions (tides and non-estuarine freshwater input) at Wuhaozhuang were similar to those in the mudflats at the salt marsh site and cordgrass transplants performed well there, transplanting artifacts should not comprise our results.

In conclusion, our results indicate that cordgrass invasions in the Yellow River Delta are likely to be limited to tidal flats and low estuarine marshes where flooding is frequent and soil salinity is relatively low. Salt marshes and high estuarine marshes dominated by native *S. salsa* and *T. chinensis* plants are unlikely to be invaded due to harsh physical conditions. These findings have important implications for understanding *Spartina* invasions in northern China [43], [46] and on other coasts with similar biotic and abiotic environments [31], [33], [47].

## Supporting Information

Table S1 **Cordgrass performances (means±1SE) in different zones at the salt marsh site in 2010.**
(DOC)
