# Supplementary material for: Physical Stress, Not Biotic Interactions, Preclude an Invasive Grass from Establishing in Forb-Dominated Salt Marshes
Source: PLoS One. 2012 Mar 14;7(3):e33164. doi: 10.1371/journal.pone.0033164 (PMC3303875; doi:10.1371/journal.pone.0033164)
Supplement: Table S1 — Cordgrass performances (means±1SE) in different zones at the salt marsh site in 2010. (DOC) [file pone.0033164.s001.doc]

**Supporting information**

**Table S1** Cordgrass performances (means ± 1SE) in different zones at the salt marsh site in 2010.

| Response variable | Mudflat | Low marsh | | High marsh |
| --- | --- | --- | --- | --- |
|  |  | No neighbors | With neighbors |  |
| Number of stems | 1.07±1.07 | 2.88±2.01 | 2.63±2.11 | 0 |
| Maximum stem height (cm) | 1.60±1.60 | 7.63±4.42 | 6.25±4.30 | 0 |
| Number of inflorescences | 0.13±0.13 | 0.25±0.25 | 0.50±0.50 | 0 |
| Biomass (g) | 0.24±0.24 | 0.88±0.56 | 0.59±0.48 | 0 |
